# Pediatric Traumatic Canalicular Lacerations: Characteristics and Prognostic Factors

**DOI:** 10.1155/joph/8582651

**Published:** 2025-04-24

**Authors:** Ran Zhao, Shaolei Han, Yuan Wen, Tingting Wang, Yiming Fan, Jianjie Wang, Yifan Wang

**Affiliations:** ^1^Department of Ophthalmology, Hebei Medical University, 361 Zhongshan East Road, Chang'an, Shijiazhuang 050017, Hebei, China; ^2^Department of Ophthalmology, Hebei Eye Hospital, 399, Quanbei East Street, Xingtai 054001, China

**Keywords:** canalicular laceration, characteristics, pediatric, prognostic, traumatic

## Abstract

**Background:** To elucidate the epidemiologic and clinical characteristics of pediatric traumatic canalicular lacerations treated at a tertiary hospital and analyze the prognostic factors of influencing functional outcomes.

**Methods:** This retrospective review included all pediatric patients who sustained a primary canalicular laceration at Hebei Eye Hospital between January 1, 2013, and December 31, 2022. Data on patient demographics, mode of injury, and surgical outcomes were collected through detailed chart review. The prognostic factors of affecting functional outcomes were assessed using Fisher's exact test for categorical variables.

**Results:** The study included 89 pediatric patients (66 males and 23 females) with a mean age of 7.26 years. There were 65 patients with lower canalicular lacerations, 19 patients with upper lacerations, and 5 patients with concurrent lacerations. Right eye damage was observed in 51 patients and 38 with damage to the left eye. The most common cause of injury was scratches caused by sharp objects (52.8%), followed by electric bicycle accidents (18.0%), falls (18.0%), and dog bites (7.9%). Statistically significant prognostic factors for functional outcomes included the location of the laceration (*p*=0.009), mode of injury (*p*=0.045), and the time interval from injury to surgical repair (*p*=0.032).

**Conclusions:** In this study, key prognostic factors for pediatric canalicular lacerations included laceration location, injury mechanism, and delay in surgical repair. Variables such as age, gender, affected side, stent type, and removal timing did not significantly impact outcomes. Increasing hazard awareness of the caregiver, enhancing public education, and implementing preventive measures to reduce injury incidence are crucial for prevention.

## 1. Introduction

Eyelid lacerations represent nearly one-fifth of pediatric ocular-related hospital admissions [1, 2]. Among these cases, canalicular lacerations constitute approximately 16% [3]. These injuries typically result from either penetrating or blunt trauma to the eyelids and periorbital area [4, 5]. Disruption of the lacrimal drainage system can lead to posttraumatic epiphora, which can significantly affect quality of life and mental health [6, 7].

Canalicular lacerations can occur across all age groups, but they are more commonly reported in children and young adults [8–11]. Previous studies have identified predictive factors that influence outcomes after canalicular laceration repair in both adults and children [10, 12–17]. However, children differ from adults in terms of exposure to distinct trauma mechanisms and a generally slower or diminished regenerative capacity of the skin with age [18]. There has been limited discussion focusing exclusively on the pediatric population. Although reports by Agarwal et al. [10] and Huang and coauthors [11] provide relevant insights, their findings cannot be directly extrapolated to our region due to differences in demographics, lifestyle, and socioeconomic status.

Therefore, this study aimed to delineate the clinical characteristics and prognostic factors of pediatric traumatic canalicular lacerations in our specific population and suggest necessary changes to the current clinical practice patterns.

## 2. Materials and Methods

This retrospective study reviewed the medical records of pediatric patients who underwent primary canalicular laceration repair at Hebei Eye Hospital in North China over a ten-year period from January 1, 2013, to December 31, 2022. The study adhered to the principles of the Declaration of Helsinki and was approved by the Institutional Research Board of Hebei Eye Hospital. The ethics approval code is 2019KY010.

The inclusion criteria were patients under 18 years of age, underwent canalicular laceration repair, and had at least one follow-up assessment. The exclusion criteria were nonprimary laceration repair, pre-existing epiphora, and incomplete documentation. Data collected included patient age, gender, affected side, location of the laceration, mode of injury, time lag from injury to repair, type of stent used, timing of stent removal, and the presence or absence of epiphora after stent removal. Caregivers were contacted by phone to provide detailed information about the patients' epiphora, such as preinjury presence, precipitating conditions (wind, sun, and cold), and the epiphora was intermittent or constant.

**Figure 1:**
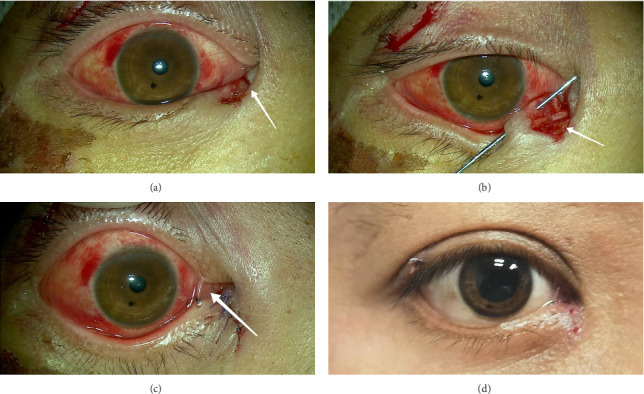
(a) A 14-year-old boy broke his right eye while riding an electric bike. The arrows in the figure show the broken lower lacrimal canaliculi. (b) Preoperative photograph showing right lower canalicular laceration. The arrows in the figure show the broken lower lacrimal canaliculi inserted into the lacrimal probe. (c) Postoperative appearance of the new RS stent in situ. The arrow shows the new RS stent in place. (d) The patient was photographed 7 days after surgery.

The surgeries were performed under local or general anesthesia by an experienced oculoplastic surgeon by using a surgical microscope. The procedure involved exploration of the proximal canaliculus, dilation of the lacrimal punctum, and insertion of a bicanalicular silicone stent (Jinan Runshi Medical Devices Co., Ltd., Shandong Province, China) or conventional stent. New RS stent is a bicanalicular silicone stent with a metal stent. When the stent was inserted into the distal nasolacrimal duct, the metal probe was withdrawn from the stent. The blue mark in the center of the canal at the inner canthus indicates that the stent is in place. Conventional stents have two probes with a ring-shaped silicone double-lacrimal-point drainage stent. A probe was inserted into the lacrimal punctum, the temporal side of the tear duct, the nasal side, lacrimal sac, and nasolacrimal duct in turn, reaching the inferior nasal meatus. A retractor was used to hook the lower end of the probe in the inferior nasal meatus and then lift it. Then we removed the probe. Another probe was inserted in the same way, then hooked out, and removed. The two ends of the silicone stent were combined and ligated, with the remaining end placed in the nasal vestibule. The lacerated canalicular ends and the surrounding tissues were sutured intermittently using 6-0 absorbable sutures (Ethicon, polyglactin 910), and the eyelid margin was repaired similarly (Figure 1). Patients were prescribed topical antibiotics (0.3% levofloxacin eye drops, Hebei Chuangjian Pharmaceutical Co., Ltd.) and steroids (0.1% fluorometholone eye drops, Santen Pharmaceutical Co., Ltd.) four times daily postoperatively for 2 weeks to prevent infection and adhesion formation. Patients were advised against rubbing their eyes to avoid early stent displacement. Stent removal was planned for 3 months post-operation. Conventional stents were retrieved from the nose in the operating room. The bicanalicular silicone stent was directly removed from the outpatient department.

Anatomical success was defined as patency on syringing. Functional success was defined as the absence of epiphora at the last follow-up visit even under environmental stressors. Functional success was the primary outcome measure for statistical analysis. Patients were categorized into epiphora and no epiphora groups.

Data analysis was conducted using the SPSS software Version 23. Descriptive statistics were described using the frequency and percentage distributions. Our study was a retrospective case-control study. We grouped based on whether the functions were successful or not and the data were binary variable data. So, chi-square test was chosen to assess the prognostic factors affecting functional outcome for categorical variables. Our total sample size was all greater than 40. When there was at least one cell with an expected count less than 5 or when there was at least one cell whose expected count was less than 1, we chose Fisher's exact probability statistic.

## 3. Results

From 2013 to 2022, of the 148 patients who underwent canalicular laceration repair, 89 pediatric patients met the inclusion criteria and were enrolled in the study.  Table 1 provides demographic details and clinical characteristics of the patients.

The majority were males (*n* = 66; 74.2%) with a mean age of 7.26 years (range, 1–17 years). Right eye damage was observed in 51 patients (57.3%) and none of the cases involved bilateral canalicular lacerations.

Most patients (94.4%) had isolated canalicular lacerations, while 5 patients (5.6%) presented with concurrent upper and lower canalicular lacerations. The injuries more commonly affected the lower lacrimal canaliculi (77.4%) than the upper lacrimal canaliculi (22.6%). The leading cause of canalicular lacerations was scratches caused by sharp objects (47 patients, 52.8%), followed by electric bicycle accidents (16 patients, 18.0%), falls (16 patients, 18.0%), dog bites (7 patients, 7.9%), altercations (2 patients, 2.2%), and sports-related incidents (1 patient, 1.1%). Most patients (60.7%) experienced penetrating trauma including scratched by sharp objects and dog bites.

All 89 patients had at least one follow-up assessment to evaluate postoperative epiphora, even under environmental stressors. Three patients (3.4%) underwent surgical intervention more than 48 h after injury, and two of these experienced postoperative epiphora. All other surgeries occurred within 48 h, and there were no intraoperative complications.

The majority of the repairs (61.8%) utilized a new RS stent and 34 patients received conventional stents. The stent removal interval for most patients was 3 months (96.6%). There was only one instance of premature stent loss, and two stents were in place for less than 3 months. All the stents were successfully removed from the outpatient department. In total, 79 patients achieved functional success, whereas 10 patients experienced postoperative epiphora. The functional success rate was 88.8%, and it was defined as a complete lack of epiphora. We had 80 patients with anatomical success, and the anatomical success rate was 89.9%. In all patients with persistent epiphora, syringing of lacrimal passages were non-patency three months after the operation in 9 patients. Using chi-square test for categorical variables, the prognostic factors identified for pediatric traumatic canalicular lacerations included the location of the laceration (*p* = 0.009), mode of injury (*p* = 0.045), and time lag from injury to repair (*p* = 0.032). There was no significant association between functional success and age (*p* = 0.723), gender (*p* = 0.443), affected side (*p* = 1.000), type of stent (*p* = 0.172), or timing of stent removal (*p* = 1.000). The results are presented in Table 2.

## 4. Discussion

In this study, we evaluated the clinical characteristics and prognostic factors of pediatric traumatic canalicular laceration. The patient cohort predominantly comprised males, with injuries primarily affecting the right eye. Most cases involved isolated canalicular laceration, and the mean patient age was 7.26 years. The leading cause of these lacerations is scratches caused by sharp objects. Most patients underwent surgical intervention within 24 h of injury, and the stents were typically removed after an average duration of 3 months. Notably, our study demonstrated a functional success rate of 88.8% among the patients.

In 2007, Salvin [19] reported that 35% of eye injuries in the United States occurred in children younger than 17 years, and 18% in children under the age of 12 years. This heightened risk may be attributable to the more active nature of children compared to adults, as well as their lack of awareness of the potential dangers. Our results indicated that among the 89 patients with pediatric canalicular lacerations, 66 (74.1%) were male. Numerous reports had consistently demonstrated a male predominance in canalicular lacerations [20–23], which may be attributed to a greater propensity for high-risk behavior in males [24]. Therefore, the caregivers of boys should pay more attention to avoid the risk of trauma in boys, especially eye trauma.

In our study, the majority of patients had right-eye injuries, likely due to the tendency of most children to be right-handed. Notably, none of the patient-related factors, such as age (*p*=0.723), gender (*p*=0.443), or affected side (*p*=1.000), predicted the functional outcomes. Furthermore, the type of stent used and the timing of stent removal were not associated with postoperative functional success, which is consistent with findings from other studies [25–27].

In contrast to studies involving adults and children, the most common mode of injury in our study was direct trauma, including scratches caused by sharp objects (47 patients, 52.8%) and dog bites (7 patients, 7.9%), followed by electric bicycle accidents (16 patients, 18%). These findings are consistent with those of studies that specifically focused on the pediatric population [10]. However, the causes of direct trauma are varied depending on the region, ethnic population, and practice pattern. For example, a study conducted in Philadelphia [22] found that dog bites were a more prevalent mechanism of direct trauma in children, especially among those younger than 10 years. It was similar to the result reported by Huang et al. [11]. Additionally, some cases have noted a predominance of hooked objects in causing canalicular lacerations [3]. In Saudi Arabia [13], metallic clothing hangers were the most common mode of injury, potentially linked to traditional attire, such as sari and blouse.

In our study, the objects that caused the most direct trauma were sharp iron objects. Given that canalicular lacerations may be associated with social determinants, it is essential to emphasize eye protection during activities such as play in community health education initiatives. Furthermore, we found that blunt trauma negatively affected the functional repair prognosis. Therefore, patients presenting with blunt trauma should receive intensive treatment to optimize functional recovery.

In our series of 89 cases, the majority presented with isolated canalicular lacerations (94.4%). The incidence of bicanalicular lacerations was 5.6%, which is lower than the rates reported by Singh [12]. This discrepancy may be attributable to the inclusion of adults in their studies, and the primary cause of injury was indirect trauma, which results in more severe wounds.

There is a growing consensus that all canalicular lacerations require repair [28], and the earlier belief that monocanalicular lacerations did not necessitate reconstruction is no longer supported [29]. In line with this conclusion, Ramsay et al. and Singh et al. [1, 2] emphasized that uninvolved canaliculi are always at a risk of future injuries. Our study found that the bicanalicular lacerations were significantly associated with poor prognosis (*p*=0.009). Therefore, we recommend the reconstruction of monocanalicular lacerations to prevent subsequent bicanalicular lacerations, thereby mitigating the risk of unfavorable outcomes.

Currently, there are differing opinions regarding the necessity of performing surgical repair within 48 h to ensure optimal functional outcomes. One viewpoint suggests that operative repair should occur within 24–48 h [3], as prolonged delays can lead to scarring and increased stenosis and complicate the identification of the canalicular ends. Consequently, reconstruction within 48 h has become standard practice. Similarly, Chu et al. [30] reported that delayed surgery (beyond 48 h) yielded a success rate comparable to that of early repair (within 48 h), particularly when performed by an experienced oculoplastic surgeon. Huang et al. [11] also found no association between postoperative complications and delayed repair. In our study, most patients underwent surgical repair within 48 h of injury. Notably, we identified three patients (3.4%) who underwent surgical intervention more than 48 h post-injury; of these, two patients developed postoperative epiphora. The finding was statistically significant. Therefore, we recommend that surgical intervention be performed as soon as possible within 48 h.

Our study had several limitations. All surgeries were performed by qualified attending physicians or associate chief physicians in a controlled operating room environment. Therefore, the influence of individual surgeon skills and the surgical environment on the success rate of functional repair was not evaluated. Additionally, our study was retrospective and the sample size was relatively small. Conducting research at a single trauma center limits the generalizability of our findings. A larger multicenter study would be beneficial in establishing the prognostic factors associated with pediatric canalicular lacerations.

## 5. Conclusions

The majority of patients in our study were male, with right eye injuries and isolated canalicular lacerations, with an average age of 7.26 years. The most common cause of canalicular lacerations was penetrating injury caused by sharp objects. Our analysis revealed no significant association between functional success and factors such as age, sex, affected side, type of stent, or timing of stent removal. Instead, the key prognostic factors identified at our institution were the location of the laceration, mechanism of injury, and time delay from injury to surgical repair. Given these findings, it is essential to enhance public awareness of the specific hazards that can lead to canalicular lacerations in children. Public education initiatives should focus on prevention strategies, emphasizing the importance of raising safety awareness among caregiver and implementation of preventive measures to reduce the incidence of such injuries.

## Figures and Tables

**Table 1:** Demographic details and clinical profile of children.

Characteristics	Value *N* (%)
Mean	7.26

*Gender*	
Male	66 (74.2)
Female	23 (25.8)

*Affected sides*	
Right eye	51 (57.3)
Left eye	38 (42.7)

*Time lag from injury to repair*	
Early (< 48 h)	86 (96.6)
Delayed (≥ 48 h)	3 (3.4)

*Mode of injury*	
Penetrating	54 (60.7)
Blunt trauma	35 (39.3)

*Mode of injury*	
Scratches from sharp objects	47 (52.8)
Electric bicycle accidents	16 (18.0)
Falls	16 (18.0)
Dog bites	7 (7.9)
Altercations	2 (2.2)
Sports-related	1 (1.1)

*Location of laceration*	
Lower eyelid	65 (73.0)
Upper eyelid	19 (21.4)
Upper and lower eyelids	5 (5.6)

*Type of stent*	
New RS stent	55 (61.8)
Conventional stent	34 (38.2)

*Timing of stent removal*	
≥ 3 months	86 (96.6)
< 3 months	3 (3.4)

**Table 2:** Association between functional success rate and location of laceration, mode of injury, and time lag from injury to repair.

Variables	Functional success *N* (%)	Functional failure *N* (%)	*p* value
*Gender*			
Male	57 (86.4)	9 (13.6)	0.443
Female	22 (95.7)	1 (4.3)	

*Affected sides*			
Right eye	45 (88.2)	6 (11.8)	1.000
Left eye	34 (89.5)	4 (10.5)	

*Time lag from injury to repair*			
Early (< 48 h)	78 (90.7)	8 (9.3)	0.032
Delayed (≥ 48 h)	1 (33.3)	2 (66.7)	

*Mode of injury*			
Blunt trauma	28 (80)	7 (20)	0.045
Penetrating	51 (94.4)	3 (5.6)	

*Location of laceration*			
Lower	63 (96.9)	2 (3.1)	0.009
Upper	12 (63.2)	7 (36.8)	
Upper and lower	4 (80)	1 (20)	

*Type of stent*			
New RS stent	51 (92.7)	4 (7.3)	0.172
Conventional stent	28 (82.4)	6 (17.6)	

*Timing of stent removal*			
≥ 3 months	76 (88.4)	10 (11.6)	1.000
< 3 months	3 (100)	0 (0)	

## Data Availability

The data that support the findings of this study are available from the corresponding authors upon reasonable request.
